# An observational comparison of FACT and ACT in the Netherlands and the US

**DOI:** 10.1186/s12888-022-03927-x

**Published:** 2022-05-03

**Authors:** Koen Westen, Patrick Boyle, Hans Kroon

**Affiliations:** 1 Avans University of Applied Sciences, Breda, The Netherlands; 2 CCAF, Utrecht, The Netherlands; 3 Reinier van Arkel, ’s, Hertogenbosch, The Netherlands; 4 Center for Evidence Based Practices, Mandel School of Applied Social Sciences, Case Western Reserve University, Cleveland, OH USA; 5 Department of Mental Health Care and Participation, Trimbos Institute, Utrecht, The Netherlands; 6 Department of Social and Behavioral Sciences, Tranzo Scientific Center for Care and Welfare, Tilburg University, Tilburg, The Netherlands

**Keywords:** Severe mental illness, Flexible assertive community treatment, Assertive community treatment

## Abstract

Assertive Community Treatment (ACT) is a well-defined service delivery model for the care and treatment of the most severely mentally ill in the community with American origins. The Dutch have adapted the model in order to accommodate a broader range of needs and allow more flexible implementation. Functional Assertive Community Treatment (FACT) provides the intensity of care needed to help participants sustain life in the community as well as continuity of care over time for many vulnerable client populations.

## Introduction

The process of deinstitutionalization led to an 80% decline of the inpatient population in American inpatient mental health institutions from 1965 onwards. Unfortunately, to achieve good quality of life and inclusion for people with serious mental illness, the closing down of institutions was not enough. Outpatient services in America were not systematically developed to deliver care to all people with severe mental illness (SMI) being released from inpatient institutions to help them integrate into society, as intended; services were not planned sufficiently to address the need [1]. Stein and Test [2] envisioned the positive impact of community living and the negative impact of hospitalization and piloted a program, a precursor of Assertive Community Treatment, namely the Program of Assertive Community Treatment (PACT). They treated and trained clients in community living and worked closely with community resources. Its core ingredient, Assertive Community Treatment (ACT), became the name most commonly used throughout the country [3]. Still ACT-teams deliver mental health services in the community to people with the most severe of mental illnesses. ACT is an integrated, multidisciplinary service delivery model (staffed with expertise in case management, psychiatry, nursing, peer support, employment specialists and substance ab use specialists), and time-unlimited services. ACT is also characterized by a team approach, in vivo services, small, shared caseloads, flexible service delivery based on individualized consumer needs, a fixed point of responsibility for all services within the ACT team, and 24/7 crisis availability [4]. Research has shown ACT to be effective in the U.S., reducing treatment costs, reducing psychiatric hospitalization and improving outcomes on several factors [5]. The Patient Outcomes Research Team (PORT) found that people in America who might benefit from ACT often did not receive this intervention [6]. Organizations see ACT as a fundamental element in a mental health service system. The Centers for Medicare and Medicaid Services (CMS) authorized ACT as a Medicaid-reimbursable treatment. ACT has been endorsed as an essential treatment for serious mental illness in the Surgeon General’s Report on Mental Health [7]. However, states have been slow to fully implement the model to meet consumer needs over the past 20 years due to inadequate staff and funding resources to cover and sustain the costs of ACT teams in America [3].

As stated, results inside the U.S. are not consistent [8], yet, ACT-teams have been developed throughout Western Europe, Scandinavia, Australia, Canada, and among other countries as mental health authorities have realized the need for assertive outreach services for this vulnerable population when closing inpatient psychiatric units. Currently, the program stands at a crossroads, strained by the principle of adherence to a long-standing operational framework, on the one hand, and calls to adjust to an environment of changing demands and opportunities on the other hand [3].

A few years after the introduction of ACT in The Netherlands during the National Evidence Based Practices Movement [9], Dutch mental health professionals stood on those same crossroads and called for adjustment to the ACT-model. These adjustments needed to address two main concerns with the model. Firstly, it seemed difficult to develop ACT-teams in rural areas and less densely populated areas. Secondly, professionals became aware of the narrow definition of the target group for ACT and wanted to provide the ACT ingredients to all people with severe mental illness. These two topics have been addressed in American literature on ACT as well. For instance, ACT was evaluated and found to be efficient in urban, densely populated areas [5] and less suited for rural settings [10]. Rural areas do not need the intensity of care all the time and need to explore ways to deliver services to all people with SMI, not just the most severe as in ACT. As indicated in earlier writings about ACT in America, it has been difficult to develop such teams in rural areas [10, 11].

This led the Dutch to introduce Functional, later Flexible Assertive Community Treatment (FACT) in 2004 [12], as an adapted and expanded model of Assertive Community Treatment [2]. Just as ACT, FACT combines the principles of team case management with delivering services to a shared caseload as needed, together with all the other assertive and outreach services within one team. The main difference between ACT and FACT is that in FACT the upscaling and downscaling of care has been structured and systematically organized. Due to this process, clients receive team case management from one case manager coordinating treatment *or* assertive outreach services from the team as a whole, being part of a shared caseload [12, 13]. The number of FACT-teams increased rapidly to 300 certified teams in 2018 [14]. Along the way, teams in the Netherlands started using FACT for subpopulations of people with SMI, including youth, people with intellectual disabilities and people with a forensic title. Delivering treatment as a regular FACT-team in times of crisis, treatment and recovery helps continuity of care and prevents dropout [15]. FACT has also shown to reduce (long-term) admissions for adult patients in the Netherlands [15], the UK [16] and Denmark [17]. FACT always delivers integrated treatment for people with interrelated problems on multiple domains of life.

### Raising doubt

At first a comparable model fidelity scale was created for FACT in 2008, adapting the Dartmouth Assertive Community Treatment Scale which was introduced in 1998 [18]. Research found an association between (F)ACT model fidelity and client outcomes [13, 15, 19–21], so strict conformation to the model was promoted. Recently the FACT-scale 2017 replaced this initial version [22]. Its shape has shifted from a standardized fidelity scale using a quantitative questionnaire to an appreciative audit with a short list of closed questions and a large qualitative area using different main topics [23] to keep up with myriad adaptations [10] of FACT and still be able to access fidelity. Researchers in the US developed a successor to the DACTS as well and created the Tool for Measurement of ACT [24] adding quantitative, recovery-oriented items to the scale. ACT and, later on, FACT share a history together and have had similar struggles in developments during their existence. There would not be FACT without the years of experimentation and research evaluation done by ACT-specialists. Bond and Drake [25] compared ACT and FACT as being similar entities. Recent changes in both model fidelity scales and challenges for both models during implementation around the world has led us to think differently. Though FACT emerged from ACT, a new comparison applying this perspective will help the practical application in theoretical discussions going on in the field of community mental health. Especially now FACT seems to gain more and more popularity around the world [26]. We conducted an observational comparison during a two-weekly observational study in Dutch FACT-teams and multiple reflective conversations with experts from the US and the Netherlands. During the process literature on ACT and FACT and all fidelity scales were analyzed. Table 1 identifies several important qualitative differences between the scales. It will help the reader identify general, but not detailed, differences between the models made in a time of transition from one model fidelity scale (DACTs) to the other (TMACT) for the ACT-model.

**Table 1:** Comparisons of Elements of ACT and FACT between America and the Netherlands

Elements	American ACT-team	Dutch FACT-team
Admission criteria	Requires a combination of patterned psychiatric hospitalizations, emergency services, substance abuse and/or criminal justice system involvement, homelessness, medication non-adherence, and not benefitting from usual mental health services • Criteria is carefully applied to the team’s client constellation to match insurance reimbursement qualifications • ACT criteria focuses only on the clients with the most severe and persistent problems (projected at 10–20%).	Includes 100% of people with severe mental illness (both the 20% for whom ACT was initially intended and the other 80% of that population, who need at times less intensive treatment and support) • No one with SMI is excluded from services, thus enhancing proactive intervention rather than waiting for incidents that trigger authorization for ACT services to begin • Services are provided to a broader group of people outside of American ACT-team criteria such as those having Borderline diagnosis, autism, developmental disability, and adolescents
Team Structure	• Small caseload (10:1) of about 100 clients/team on average; size of team may vary • Team members: ideally 90% of clients have contact with more than 1 team member within 2 weeks, team meets at least 4 × per week to discuss each client’s care, supervisor provides at least 50% direct care, less than 20% staff turnover within 2 years, 95% full staffing within past year, including psychiatrist/ psychiatric prescriber, nurse, substance abuse specialist, vocational/employment specialist; team size and diversity is sufficient for caseload coverage, explicit admission criteria (noted above), low intake rate, ideally 80% or more of contacts in community •Full responsibility for services	• Small caseload (15:1) of about 200 clients/team on average; size of team may vary • Team members: Flexible care (scaling of intensity up and down), team approach provides contact with at least 4 disciplines, daily FACT board meeting evaluates and directs interventions, over 70% community outreach; at least 50% of team members have at least .78 FTE with the team, including psychiatrist, psychologist, nurse, employment, social work, employment specialist, peer support, physical health, addiction, Mild Intellectual Disorder expertise, assigned coordinating and monitoring roles • Full responsibility for services presumed and not measured
1. Flexible Care	Frequency of contact per week is determined by the team during ACT-team meetings (optimally daily meetings), as needed, from daily contact to less frequently. Contact with the client support system in the community by the team is also expected and monitored • Clients are appear to remain on the ACT-teams for a long time (several years) and transferred off the team for ongoing care once determined to be more self-sufficient where care is rendered by other case managers not with the ACT-team • Client support system in the community is not often successfully developed	Care is scaled up or down during the daily FACT-board meetings by the team and in collaboration with the network partners also providing care (includes General Practitioner, district social service team, inpatient care) in concert with stage of recovery as reflected in treatment plan • Flexibility allows the intensity of services to range from inpatient care to daily care and then transferred to community based district social service teams and other social supports over time. Intensive care appears to be needed for some (about 10%) on a temporary basis, for another 10%, longer term
2. Personal Domain	Individualized treatment plans and individualized treatment are required that reflect the client’s goals and is reflective of assessed issues. Consumer choice guides treatment in all ways, including the location of housing, the nature of general health care, assistance with financial management, daily living skills to be taught, medication support, and the nature of substance abuse treatment • Does not explicitly by designed expectation address client identity issues, staff stigma and hopeful attitude	Central to this domain is the whole team acknowledging the client’s individuality, the client’s own strength as its starting point, perceiving the client’s struggle with their cultural, sexual and spiritual identity and emotions such as grief and sorrow, combating stigmatization by the team and self-stigmatization by the client, taking risks and having a hopeful attitude while using hopeful language oriented towards an open and positive picture of the future
3. Social Domain	Consumer choice guides treatment in the location of housing, the nature of general health care, assistance with financial management, daily living skills, medication support, the nature of substance abuse treatment and other chosen issues identified by the treatment team and client • Expects connection with informal support system (family, friends, and proprietors) though by superficially counting number of contacts • Does not explicitly by designed expectation address loneliness, leisure, safe living, involvement of other professional network partners	Formulating and achieving goals of the client’s roles within three domains are evident: 1) 'self-care and living' such as finding housing, preventing homelessness and sorting out financial issues; other team members may focus on loneliness, pathways to work or training, self-care or safe living; 2) ‘social network’; and 3) 'work and leisure’. Interventions are prepared in conjunction with the client, their family and the team’s professional network partners • Connection with a wider range of social contacts fosters community integration
4. Symptomatic Domain	Consumers have standardized, high quality assessments that includes: history and treatment of medical, psychiatric, and substance use disorders, current stages of all existing disorders, vocational history, any existing support network, and evaluation of biopsychosocial risk factors. The information is comprehensive across all assessment domains and updated at least annually • ACT-model does not explicitly address physical health issues nor teaching strategies that address skill development	The team seeks to achieve the highest possible level of mental and physical well-being for the client by implementing a system in which screening, diagnostics, treatment interventions and evaluation all take place in accordance with the most recent research findings from psychiatry, medication, physical health and follow up, psychology, addictions, and pedagogy in teaching/transferring skills • Client education in managing the understanding and care of symptoms (disease management) is vital to sustained recovery
5. Planning & Monitoring	Process monitoring and outcome monitoring are expected of all teams. An example of a process indicator would be systematic measurement of how much time individual case managers spend in the community instead of in the office. Process indicators could include items related to training or supervision. The underlying principle is that whatever is measured is related to implementing ACT. Supervisors and ACT leaders monitor the outcomes of ACT consumers every 3 months and share the data with ACT team members in an effort to improve services. Systematic and regular collection of quarterly to annual outcomes for their monitoring involves a standardized approach to assessing results of client interventions • Individual client based outcomes continues to be a laborious process for most teams and has largely not been standardized to address psychological and social functioning, or quality of life and recovery issues	The team has a clear treatment plan cycle, adheres to a logistical process according to good working procedures, and is responsible for the outcome of the treatment. It assumes a managing and coordinating role. Integration of the ROM (Routine Outcome Monitoring) data is part of this from a well-reasoned choice from the available standardized measuring instruments • The implementation and evaluation of the treatment and its progress take place collectively via a collaborative relationship between the team and the client, their family, the GP and the mental health worker at the GP surgery. Decision-making about treatment takes place collectively. Each party may contribute goals • At least yearly clinical Routine Outcome Monitoring (ROM) takes place for the benefit of individual strategies and treatment plans. Standardized instruments are used to measure (1) psychological and social function, (2) needs and (3) quality of life and recovery
6. Crisis and Safety	The team cover responsibility for 24-h crisis services and hospital admissions and discharges. Relationships with local hospitals is expected • Mechanisms for case finding or service drop outs are dependent on team member and team leader initiative not standardized • ACT-team members’ safety is often of concern	The team works from 9 until 5 and has a working alliance with an out-of-office crisis resolution team. The team has implemented policy consisting of risk assessment and the provision of evidence-based interventions relating to crisis prevention and early detection. It can be expected for the team to have a structural relationship with regional services such as the police force and other health and safety services to ensure personal safety in and around homes • The use of assertive engaging interventions, acute up scaling of care and collaboration with relevant partners are important in this regard • In its own catchment area the team can undertake targeted case finding when clients seem to drop out of care as well as untargeted cases
7. Network Collaboration	Ideally, the Process Planning and Outcome Monitoring functions are the purview of a management team that includes participants from the larger organization and community stakeholders to provide objectivity and integration with the organization’s philosophy and strategic plan • The team involved in such collaboration more often is restricted to mental health organization staff	Committed collaboration with the client’s network is of importance to ensure that control of the recovery process lies with the client and his resources of choice. The team involves the client’s (social) network (including family, general practitioners, community police, and other community health providers) in the team evaluations, supports the network with the most appropriate forms of treatment for the target group and supports and facilitates the creation of forms of self-help by the client’s personal network
8. Quality & Innovation	A Quality Improvement Committee helps guide important decisions such as penetration goals, hiring/staffing needs and sustaining the implementation by reviewing fidelity to the ACT-model, making suggestions for improvement, advocating/promoting ACT within the agency and in the community, and deciding on and keeping track of key outcomes relevant to ACT. Ideally, this function is also the purview of a wider team of participants to provide objectivity and integration with the organization’s philosophy and strategic plan. Team members are expected to participate in annual training activities to improve and sustain skills. Outside expertise is sometimes involved in this process	The FACT-team seeks to provide the highest quality of care and is open to new knowledge, initiatives and innovations. To achieve this the team has a specially designed training policy requiring at least four half-day training sessions per team member, which is set out in the Team Document. Team members continue to develop additional expertise in their field • The team regularly invites external experts or asks for their help • In addition, there is evidence the team works with a Plan-Do-Study-Act cycle to improve their quality

### Comparison of ACT and FACT

As stated by Westen et al. [23] over time some criteria (of the initial FACT-scale) lost validity. The care context has changed, and it is appropriate now to allow new qualitative initiatives and innovations. To adapt to the changing context, the Dutch have continued to evolve an essential community-based practice. American providers serve the most vulnerable people with fidelity to the DACT or TMACT. Three changes to the Dutch system has fostered its evolution: 1) nursing assignment—nurses specialized in mental health are now based in General Practitioner (GP) clinics, fostering increased integration of mental health and physical health practice. In the past, the Dutch mental health system could only downscale to GP’s and consequently FACT often remained in charge for too long, impeding recovery. Now, more mental health expertise is available at the GP clinic, allowing shared responsibility for clients’ physical health. GP care of recovering former FACT clients is a more fluid process; 2) High and Intensive Care (HIC) units – employ a multidisciplinary team (psychiatrists, nurses, psychologists, consumers) of sufficient size, and with specific training in crisis management, acute medication, and handling aggression and suicidal behavior. Even when considering hospital admission, the ambulatory recovery goals are the reference. The HIC-unit keeps admissions as short as possible and continually coordinate with clients, family and the FACT-team [27]; 3) Dutch policy change in 2015 – innovations in the service delivery system led to the development of District Social Service Teams and other municipal initiatives to foster more full civic participation and self-management. This policy change aimed for improved community integration, reduced stigma of having a mental health illness, speaking the same language, and increased ownership of the role of community members in all their citizens’ welfare focused on normalizing life. These teams share responsibility for important recovery domains such as housing, work and social contacts. Implementation has local differences and plays a significant role in the social network around clients with severe mental illness that foster recovery in various domains. In a similar fragmented mental health context in Norway, FACT-teams have shown to support closing of the gaps between organizations [28]. Additionally, the Dutch have included clients with a variety of diagnoses [29] and ages [30] that indicate a need for intensive treatment and not just adult clients with serious mental disorders.

### Implications for both models

People with serious mental illnesses have historically been underserved. While the ACT-model embraces the most severely impaired clients, it does so to the exclusion of those somewhat less impaired, those still in need of attention and whose needs may intensify at any given time. The ACT-model necessarily excludes some people with serious mental illness, largely based on state level qualifying functional and diagnostic criteria, e.g., people with Borderline Personality diagnosis. The American ACT-model requires that once a designated level of functioning is attained, the client transitions from the ACT-team since they no longer qualify for ACT-services. Though care is taken during this transition time to ensure that sufficient engagement with the new case manager has taken place (possibly over several months), this new relationship is not necessarily team-based and is ordinarily with case managers under different supervision, with much higher caseloads, and detached from the original ACT-team. Full recovery is less the focus than functionality. Given the high staff turnover in American mental health systems, it is common that clients are then reassigned to several different and new case managers within a short period of time and with less careful transition. This fragmented process creates an environment that could easily miss signs of relapse due to lack of knowledge of client needs, tenuous engagement with the client, insufficient frequency in client contact due to larger caseloads, uneducated and less developed case managers, and less than adequate multidisciplinary team integration. Transition and reassignment may actually perturb conditions of relapse with the client. The Dutch FACT-team structure and flexibility account for all of these conditions by allowing the client to stay within a (larger) team structure and receive an intensity of care from the same team over a much longer period. These differences are likely to ensure a longer and steadier recovery trajectory into more autonomous community living. Dutch FACT-teams are more inclusive of people with several conditions benefitting from intensive care, thus expanding the strengths of the ACT-model with new client populations. A goal of providing services to ALL vulnerable people is thus accomplished rather than the focus of ACT with the most severely impaired 10–20%.

Providing services for all vulnerable people in Dutch FACT-teams has been a challenge since the policy changes in 2015. FACT-teams provided integrated treatment until 2015; after 2015 a financial distinction was made between care and treatment. Professional mental health providers staff (F)ACT-teams and offer treatment. FACT-team networks include GPs and local community social networks that engage consumers beyond the end of the care continuum, allowing more full integration of care within the local community [14]. Currently this differentiation challenges the FACT-team’s ability to work in an integrated manner using a multi-agency approach and supported by the new FACT-model fidelity scale of 2017. Unfortunately, these changes led back to a more treatment-oriented approach and thus a focus on those with more severe mental illness [31]. More discharges to the GP and care-oriented teams from the municipality led to rapid deterioration of problems and a return to FACT or other specialized mental health treatment [32]. A network-orientated approach is required, embedding seamless transitions of clients and professionals. Dutch FACT-teams are experimenting using a multi-agency approach within a network of organizations or within one FACT-team, combining professionals from up to three or four different organizations.

### Differences

Several differences are apparent when comparing ACT in America with FACT in the Netherlands. These differences include who receives such services and for what duration. ACT focuses effort on those with the most serious mental illness; FACT is for all people that struggle with severe disorders that may limit their ability to live full lives in the community. FACT flexibility provides continuity of care throughout the service and into the community setting by more quickly upscaling and downscaling the care with the same team of providers. As stated earlier, the range of providers differs in important ways: ACT-teams being largely professional mental health providers and FACT-team networks including GPs and local community social networks that engage consumers beyond the end of the care continuum, allowing more full integration within the local community [14].

Perhaps more importantly, the Dutch have intentions that reflect their national norms for wellness. Every resident of the Netherlands is insured for health care, unlike the American health insurance model Medicaid. However, the various health insurers in the Netherlands also demand delivery of certain services with minimal resources. In both countries, creativity and assertiveness are necessary to adopt the model and then adapt the model to the local community’s needs. A full nationwide coverage of FACT-teams as once intended has not yet been established in the Netherlands.

Many American states have implemented ACT in recent years thanks to professional effort and due to settlements resulting from Olmstead Act lawsuits against them since they were not providing adequate mental health services to enable people with serious mental illness to live in the least restrictive environments in the community. However, few American states have proactively identified the number of people needing ACT with a plan to add sufficient teams that provide the necessarily intensive care. A lack of funding for such community based mental health programs was often a primary argument provided by the states, yet America clearly struggles with a norm of providing basic medical treatment to its entire population. There are currently about 47 ACT-teams in Ohio, an American state with nearly 12 million people; the Dutch have about 300 certified FACT-teams for a population of about 17 million people, demonstrating the significant difference in allocating such resources for people in need.

## Conclusion

Over the decades since its inception, more ACT-teams developed in America, yet the ACT-model alone is not sufficient to serve all people with severe mental illness. Its structure and functions in American teams continues much as it did in ACT’s infancy in the 1980’s and 90’s, while adding a recovery-oriented focus and evidence-based practices in recent years. In line with its national culture of pragmatism and care for all people, the Dutch have demonstrated innovation and progressive thinking. They aim to ensure that a proactive community-based network strategizes to identify, engage, and treat a wider range and variety of people with mental challenges in such a manner that maximizes their ability to live full lives in the community. FACT is attributable to ACT in many ways and both models can exist side-by-side in (larger) cities. Being able to make an educated choice between the two models within a certain context is something that will improve quality of care for all people with severe mental illness.

## Data Availability

Not applicable.
